# Traffic Impact Assessment for the Stadiums Hosting FIFA 2022 World Cup in Qatar: A Case Study

**DOI:** 10.1007/s40996-021-00723-7

**Published:** 2021-08-18

**Authors:** Shahram Tahmasseby, Padmanaban Reddipalayam Palaniappan Subramanian

**Affiliations:** 1 Qatar Transportation and Traffic Safety Center, Qatar University, Doha, Qatar; 2 Civil Engineering Department, Indian Institute of Technology Madras, Chennai, India

**Keywords:** FIFA 2022, Stadium, Master plan, Traffic modeling, Qatar, Transport accessibility strategy, Special event scenario, VISSIM

## Abstract

The State of Qatar has made extensive preparation to successfully host the upcoming FIFA 2022 World Cup, a tournament that will be held for the first time in the Middle East and the North Africa region. In preparation for this tournament, a wide-ranging operational strategy is being developed for each of the stadiums separately. This paper looks into the preparation stages of master planning and transport strategy for one of the hosting venues, which is located in Al Rayyan, Qatar. An overview of the Fédération Internationale de Football Association (FIFA) tournament, its assumptions, spatial planning, traffic modeling, Temporary Traffic Management, and the required mitigations from the transport operations perspective alongside the lessons learned are discussed in the paper.

## Introduction

Qatar is slated to host the 2022 FIFA World Cup,^1^ a first for a Middle Eastern country. The country is undergoing vast infrastructure changes to successfully host the tournament for the first time in the Middle East and North Africa (MENA) region. Qatar will be in the global spotlight and will experience a high level of transport activity during the tournament. Hence, it is necessary to understand the traffic movements in detail and plan well in advance to successfully execute the tournament. Prestigious tournaments such as the Olympic and the FIFA World cup tournaments require a comprehensive transport strategy to make the cities and the venues tournament-ready. A thoroughly researched and well-published transport strategy plan will assist the stakeholders to understand their role and their commitments to successfully deliver the tournament.

This paper discusses the approach to conduct a Traffic Impact Assessment (TIA) and the accessibility strategy for the World Cup venues. In this paper, the authors aim to share the experiences and the strategies utilized in transport master planning and operation strategies for FIFA World Cup hosting stadiums in Qatar as an overall process.

**Fig. 1:**
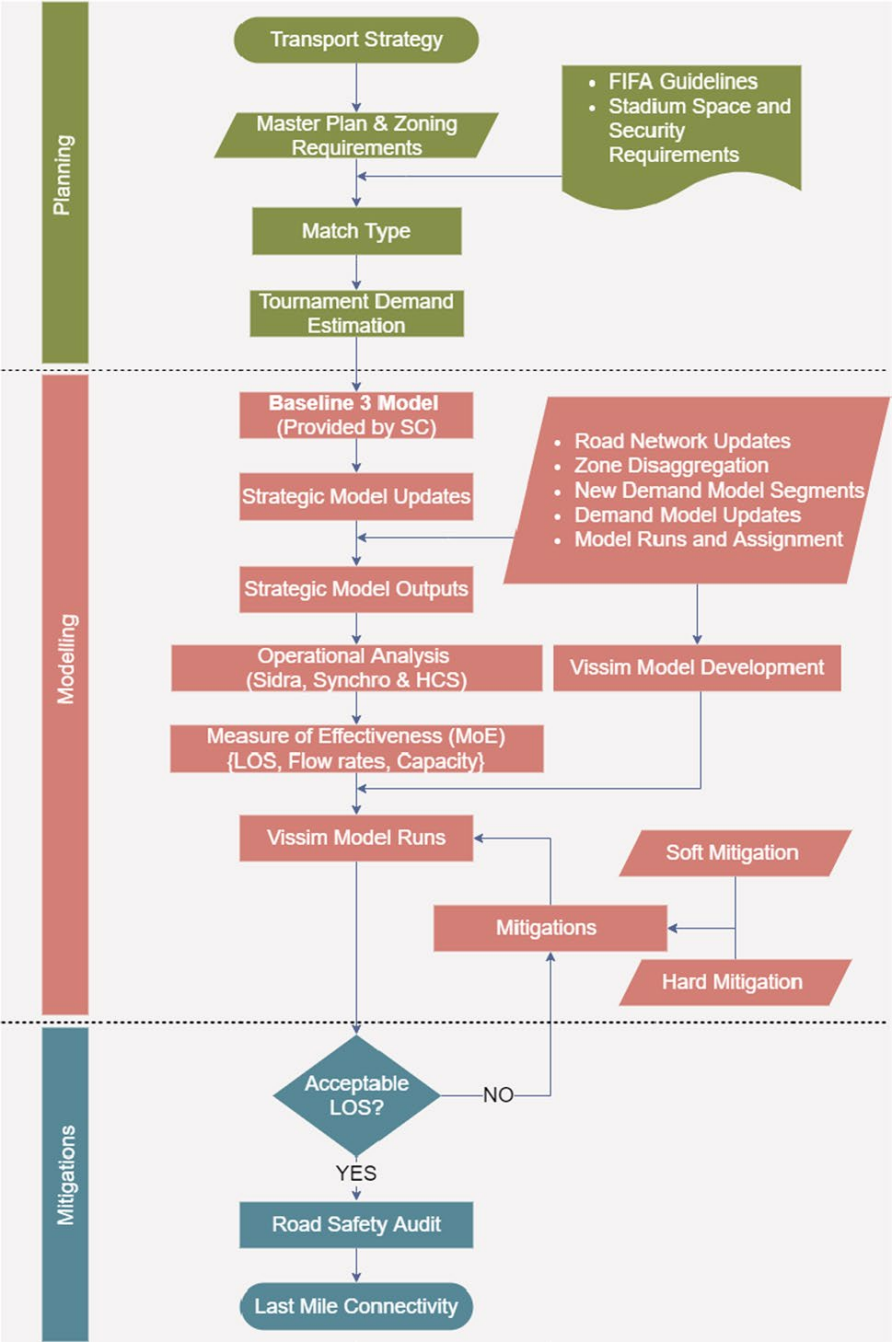
Traffic impact assessment process for venues hosting major sporting events

The following flow chart exhibited in Fig. 1 outlines the steps involved in conducting the Traffic Impact Assessment (TIA) for this study.

## Traffic Impact Assessments for Venues Hosting Major Events

Traffic Impact Assessment (TIA) deals with demand forecasting, mode choice, public transportation, master planning, and the accessibility strategy for venues hosting major events such as Olympic Games and FIFA World cup.

### Tournament Demand Forecasting and Mode Choice for Stadiums Hosting FIFA World Cup

During the FIFA World Cup, it is anticipated that fans occupy not only stadiums but also the designated venues (Fan zones) in public spaces. For instance, in FIFA World Cup (2006) hosted in Germany, 27 million fans watched the tournament at publicly designated spaces and event spots (Frank and Steets 2010) besides the stadium spectators. Consequently, the provision of diverse transportation means for such a level of demand is a sine qua non. Public transportation, passenger cars, shuttles and coaches, cabs, and taxies are the main modes of transportation in this context. Furthermore, active modes, i.e., bike, scooters, and pedestrians, are considered for access and egress, so-called last-mile connectivity, parts of travel to stadiums.

In FIFA World Cup (2006) tournament, more than half of journeys to stadiums were made by public transportation (57%), while passenger car share was 23%. Moreover, coaches and taxies partook 11% and 3%, respectively. Meanwhile, the role of active modes was considerable and reached 6% in FIFA World Cup (2006).

Passenger and travel information for public transportation, parking management, the allocation of resident protection zones and special traffic zones, the designation of bus shuttle services, and promotion of travel by bicycle were included in the FIFA 2006 action plan to facilitate transportation and provide accessibility for such huge tournament with 1.1 billion person-kilometers related to travel.

Decker et al. (2005) point out traffic and transportation as a key risk identifier among other factors such as spectator management and crowd control, public health, environmental concerns for holding special events. They indicate to (1) spectator parking areas, (2) traffic patterns, (3) access and staging areas for large numbers of emergency vehicles, (4) alterations to re-current traffic and road use, (5) traffic control, adequacy of the surrounding road network to handle the anticipated spectator vehicular traffic pre, during, and post events, (6) communication between traffic management groups and other services, (7) access and egress routes. Furthermore, they recommend that local law enforcement, transportation authorities, and public works, the local media, transit authorities, and operators should make a traffic management group and efforts on traffic planning well before the event.

Thus, accurately determining the tournament demand is a critical step toward planning an efficient transport strategy for each of the tournament venues.

To prepare for the FIFA World Cup 2022, Qatar has established the Supreme Committee for Delivery and Legacy (in short, ‘SC’), the organization responsible for developing the planning and operations and delivering the required infrastructure (Stadiums and non-competition venues).

The Al Rayyan Stadium discussed, as a case study in this paper, is one of the venues of the FIFA 2022 World Cup tournament with a net capacity of 41,000 spectators and will host the group and the knockout stage matches. There will be eight (8) venues for Qatar World Cup 2022 hosting 32 teams. The average venue capacity is 47,500 seats. The largest venue hosting the Final Match is Lusail Stadium having a capacity of 80,000 spectators. There are also multiple venues with a capacity of 40,000 seats. The case of Rayyan stadium thus is a relevant representative for transport master plan study and review in Qatar for the FIFA tournament.

To successfully deliver the tournament, an accurate estimation of trip generation, modal shares, and parking requirements in consideration of the uniqueness of each stadium is required. SC after extensive modeling and research has derived spectator profile for the tournament. The spectator profile is estimated by calibrating the existing Qatar Strategic Transport Model (QSTM) to match the tournament requirements. This resulting model is known as the Baseline 3 model and includes assumptions based on the confidential inputs from multiple stakeholders with the tournament demand, background demand, and the revised highway network.

The Baseline model also accounts for the vehicle occupancies of various user groups (spectators categorized by access levels and services offered based on the ticket value types which includes VVIP, VIP, Hospitality packages and general packages, etc.). For example, the car occupancy is considered as 2.65 persons per vehicle, whereas the taxi occupancy is 4.0 persons per vehicle, which are assumed based on factors such as past experience and local transportation studies.

As for the general arrival and departure profile, it has been assumed that 35% of the spectators will arrive during the peak hour before a match (pre-match arrival) and 83% will be departing in the peak hour after the match (post-match departure). It is also assumed that 100% of the spectators will depart from the venue within 90 min post-match. The peak hour specified here denotes the peak associated with the match which is an hour pre- and post-match which may not be necessarily the general traffic peak hour.

The profile of spectators varies according to the teams playing in a match. SC has broadly classified two types of match scenarios;

- **Qatar Match Scenario**—Qatar national team playing against another team.
- **Typical Match Scenario**—Two international teams playing against each other.

Table 1 outlines the arrival and distribution profile of the tournament demand for the above-mentioned scenarios. It should be highlighted that the spectator profile and the associated mode choice were developed by SC and are not disclosed.

**Table 1:** Spectator profile comparison

User group	Qatar	User group
Metro users	12,000	12,000
Hospitality (Cars)	6,097	2,247
Hospitality (Coaches)	631	3,710
Non-Hospitality (Cars)	18,290	9,214
Non-Hospitality (Coaches)	2,963	12,314
Taxi/shuttle riders	664	745
Spectators travel by walk	327	735
Total	40,972	40,965

### Public Transport Operational Strategy

Public transport serves as the main and preferred mode of transportation to venues for special events due to their efficient capacity and the ability to utilize the existing infrastructure without major additional constructions. Temporary bus stations could be planned in an open area closer to the stadium. If private cars are allowed near the precinct, it may result in traffic and flow breakdowns and there is a likelihood of a security breach.

For the case of FIFA (2006) in Germany and to ensure smooth and hassle-free access to the World Cup stadiums, it was planned spectators arrive at the precinct via different routes, transportation means, and service lines as specified in Federal Government Germany (2006), Stahl et al. (2004). This led to steadily assign traffic flow to the access points of the stadiums and ascertain the segregation of spectator groups while arriving and subsequently post-match.

Burke and Evans (2010) assessed proposed locations for a football stadium in Queensland Gold Coast in Australia by examining the accessibility with the focus on public transportation. They incorporated travel time including access and egress, waiting and transfer time of public transport rides. Their accessibility models demonstrated that the success of stadiums indeed depends on their convenient access to public transportation as a predominant means of transportation for spectators.

Currie and Shalaby (2011) explored strategies in transport planning for two different major events, i.e., the summer Olympic Games hosting 40,000 Olympic officials and athletes in addition to up to 8 million ticketed spectators and the Hajj/Umrah Pilgrimage in Makkah, Saudi Arabia with almost 3 million pilgrims. They synthesized lessons learned between the aforementioned two major events and elucidated the performance and implications of alternative transportation strategies for major occasions.

Hensher and Brewer (2010) applied a value-chain approach to evaluate the transportation performance of the Sydney Olympic Games. In their method, Transport Delivery System was accounted for as a significant driver in the value chain. This included measuring the efficiency of transport modes, i.e., public buses, trains, taxies, roads, and the airport and particularly having a detailed look into private bus operators.

An overview of walking and the public transport strategy from the past FIFA tournaments is given below.

#### Walking

Designated “World Cup Routes” in FIFA (2006), the distance between Friedensplatz & the Westfalenstadion, which was about 2 km, was called “Fan Mile High Street”. Cultural events, e.g., exhibitions, street arts, and music took place along the aforementioned route.^2^

The city of Johannesburg in South Africa introduced the Park and Walk during FIFA (2010) World Cup event. Spectators parked their vehicles in one of four Park and Ride facilities, i.e., Shareworld, Randshow Rd, Aeroton (all for Soccer City), and Athlone Boys (for Ellis Park), and walked to the stadium in Johannesburg. The longest distance to reach the stadium was not exceeding 2.2 Kms (Kruger^2010^).

#### Public Transportation

For FIFA (2010) World Cup in South Africa, each host city developed an integrated match-day transport plan based on their existing public transit infrastructure and capacity. Consequently, fans were encouraged to ride public transportation such as Metrorail, Metrobus Services, BRTs, and Buses from the Airport or alternatively take advantage of the tournament-specific transportation facilities, i.e., Park and Ride, Park and Walk in the game host cities.

The West Gate Transportation hub in Johannesburg in South Africa offered transportation services for spectators to reach the stadium during FIFA (2010) World Cup within walking distance. The public transport services were Rea Vaya BRT and MetroRail. Match Day ticket holders could take the Metrorail free of charge.

In Cape Town, shuttle services were established and operated between the city’s main transport hub and Cape Town Stadium in Green Point. The services were free for match ticket holders. It was operated from six hours before the game until four hours post-match with the interval of five minutes. To reach the city’s main transport hub, spectators could take shuttle service from the University of Cape Town, which was a designated Park and Ride site in the city. Besides the aforementioned Park and Ride facility, Cape Town designated two other Park and Ride sites, i.e., Camps Bay High School, Kronendal Primary School in Hout Bay.

In Brazil, Bus Rapid Transit (BRT) lines were extended to accommodate public transport demand during World Cup 2014. For instance, The Move system was planned for three corridors in the city of Belo Horizonte out of which two corridors were inaugurated in March 2014 serving the Pampulha region where Mineirão stadium is located (ITDP Belo Horizonte 2021). The Move System reduced transit times considerably in the morning peak hours, particularly by 60%. The BRT was beneficial for spectators traveling from other cities and arriving at the Belo Horizonte bus terminal. They took the rapid bus system (BRT), boarding at Central station, and then alighted at stations nearby the stadium hosting World Cup 2014.

### Master Planning and Zoning Requirements

An efficient master planning is a key to manage the transportation access requirements for respective user groups of a facility including stadiums and sports arenas. Basically, the main purpose of stadium master planning is to set as a program of works for a new stadium or renovation/expansion project. The master plan determines prerequisites and requirements, which have to be met in the stadium precinct and the surrounding area to accommodate existing and future needs.

The master plan should provide a holistic approach to enhance the stadium development to remove the potential for conflicts during the different project phases.

By incorporating transportation into master planning, vehicle access, parking facilities, temporary traffic management (TTM), route alterations, accessibility to different visiting groups, and traffic safety are addressed.

Denver City Council (2019) adopted a community planning and development strategy for the Stadium district master planning. In the master plan, mobility options, including on-street parking facilities, short interval bus services, bicycle sharing, mixed-use streets including features of bikeways, pedestrian amenities, e.g., wider sidewalks, and integration of a public transport station for regional accessibility were foreseen in the planning stage to create a walkable neighborhood and enhance transit-oriented development principles.

UEFA^3^ emphasizes the incorporation of player facilities such as dressing places, parking access. Furthermore, the aforementioned football body recommends incorporating transportation accessibility for media and broadcasting units as an integral part of modern sport in stadiums’ master planning (UEFA Guide to Quality Stadiums) (UEFA 2011).

In the context of safety planning of stadium facilities, few studies discussed the vulnerability of spectators in outdoor stadium facilities during sporting events particularly from the safety point of view. Berlonghi (1995) looked into the management and control of crowds in mega-events. His study took transportation, parking, ticket selling, and admission control alongside the size of the crowd, location of the event, day and time of event operations, crowd movement patterns, and density into consideration. Ali et al. (2011) studied the safety and health aspects of outdoor stadiums in Malaysia. They determined two objectives:

1. Identification of the outdoor stadium facility and safety measures; and
2. Evaluating the safety awareness to the associated risks for Malay spectators.

They recommended the stadium facility managers to examine the existing sport facilities’ condition within and post the sports events (particularly football matches), as the safety awareness among spectators in Malaysian outdoor stadiums seems to be high.

For the case of Al Rayyan Stadium in Qatar for FIFA World Cup 2022, the precinct master plan is divided into multiple zones for efficient and safe operations of different user groups according to the access strategy developed by the Supreme Committee. Some of the user groups who will be served by various zones are:

- VVIPs/VIPs,
- General Admission Spectators (non-hospitality),
- Hospitality Spectators,
- Workforce and Logistics,
- Information and Technology,
- Security,
- Media and Broadcast and
- Commercial Affiliate

### General Accessibility Strategy

To control the traffic movements, a Traffic Preliminary Zone (TPZ) is designated to help manage traffic flow around the stadium and divert non-essential vehicular trips away from the stadium. The TPZ includes the parking areas and the perimeter roads of the stadium, which are used for screening the vehicles and to allow or reject them entering based on access passes.

Temporary Vehicle Permit Checks (VPCs) need to be implemented at all access points to TPZ consequently.

A football stadium precinct is tremendously complex in terms of functionality. This is due to diverse multiple operations and activities which happen concurrently.

Hence, it is essential that security staff pay attention to the security plan and segregation strategy particularly for rival fan groups, which should be coordinated with the local authorities, and police patrol well before and after ticket checkpoints.

A Traffic Free Zone (TFZ), which will be enclosed within the TPZ, is planned to be accessible for selected user groups. Different types of security boundaries and control measures could be employed in the stadium during the tournament. These include security arms and barriers, segregation barriers, hostile vehicle mitigation measures (active and passive), bollards, and gates.

Any vehicle entering the Traffic Preliminary Zone has to go through a Traffic Permit Control (TPC). To enter a Traffic Free Zone, it has to pass through a Vehicle Permit Checkpoints (VPC). Any vehicle entering the Stadium Outer Perimeter must be screened at a Vehicle Screening Area (VSA). The scaling of the VSA has been based upon the forecast flow rates. VSAs are established on the Stadium Outer Perimeter to screen all vehicles requiring access (except for VVIP, Teams, and Emergency Vehicles).

## Qatar FIFA World Cup 2022 Case Study: Al Rayyan Stadium

As indicated before, the State of Qatar has made extensive preparation for the FIFA 2022 World Cup in its eight designated stadiums including Al Rayyan Stadium, which will become the new home of Qatar Stars League (QSL) outfit Al Rayyan Sports Club.

**Fig. 2:**
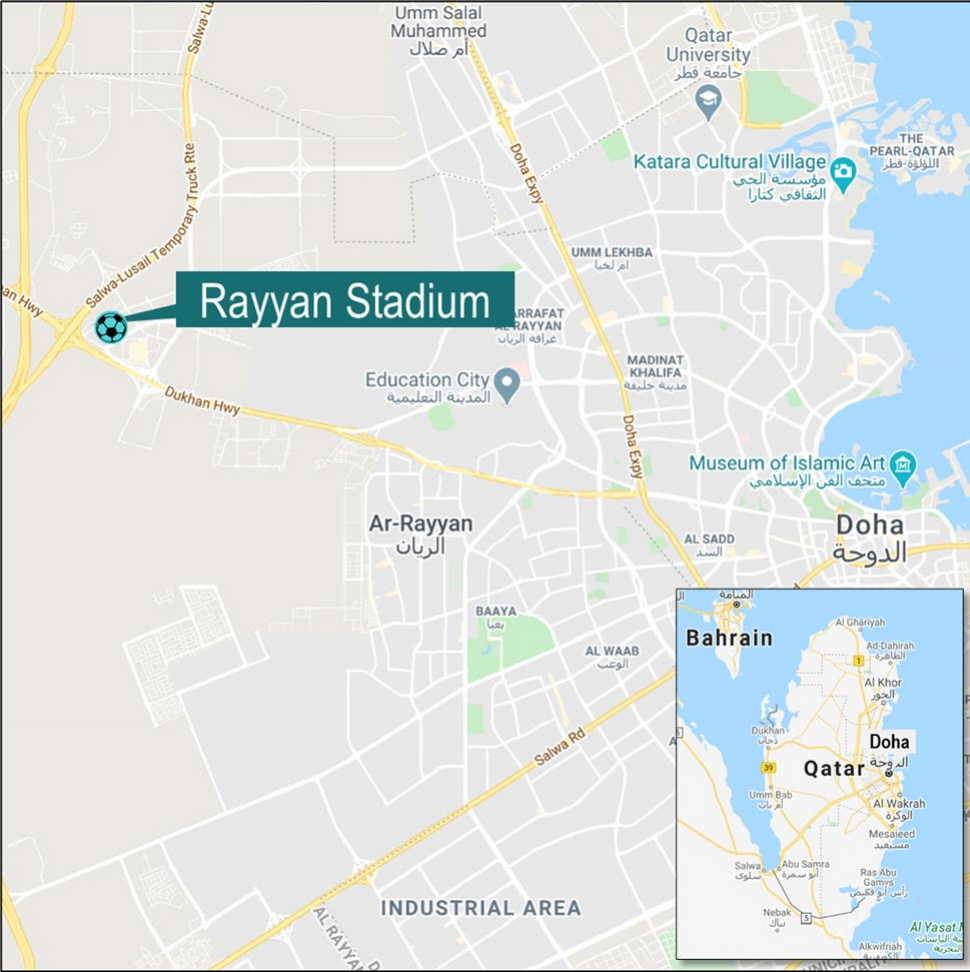
Rayyan stadium location plan

The aforementioned stadium is located in Al Rayyan Municipality, twenty (20) kilometers west of the capital with the capacity of 41,000 spectators.^4^ (Fig. 2).

The largest shopping mall in the country is located next to Al Rayyan Stadium. Hence, the transportation master plan has to deal with the arrangement of public transit for both mall visitors as well as game spectators during the tournament days. Moreover, the transportation master plan considers accessibility to the mall as well, particularly during the tournament duration.

In this section, developed strategies for access to the stadium for various user groups, travel routes, and public transit arrangements for buses and metro during the tournament will be briefly explained.

### Developing Accessibility Strategy to the Stadium Precinct for Various User Groups

**Fig. 3:**
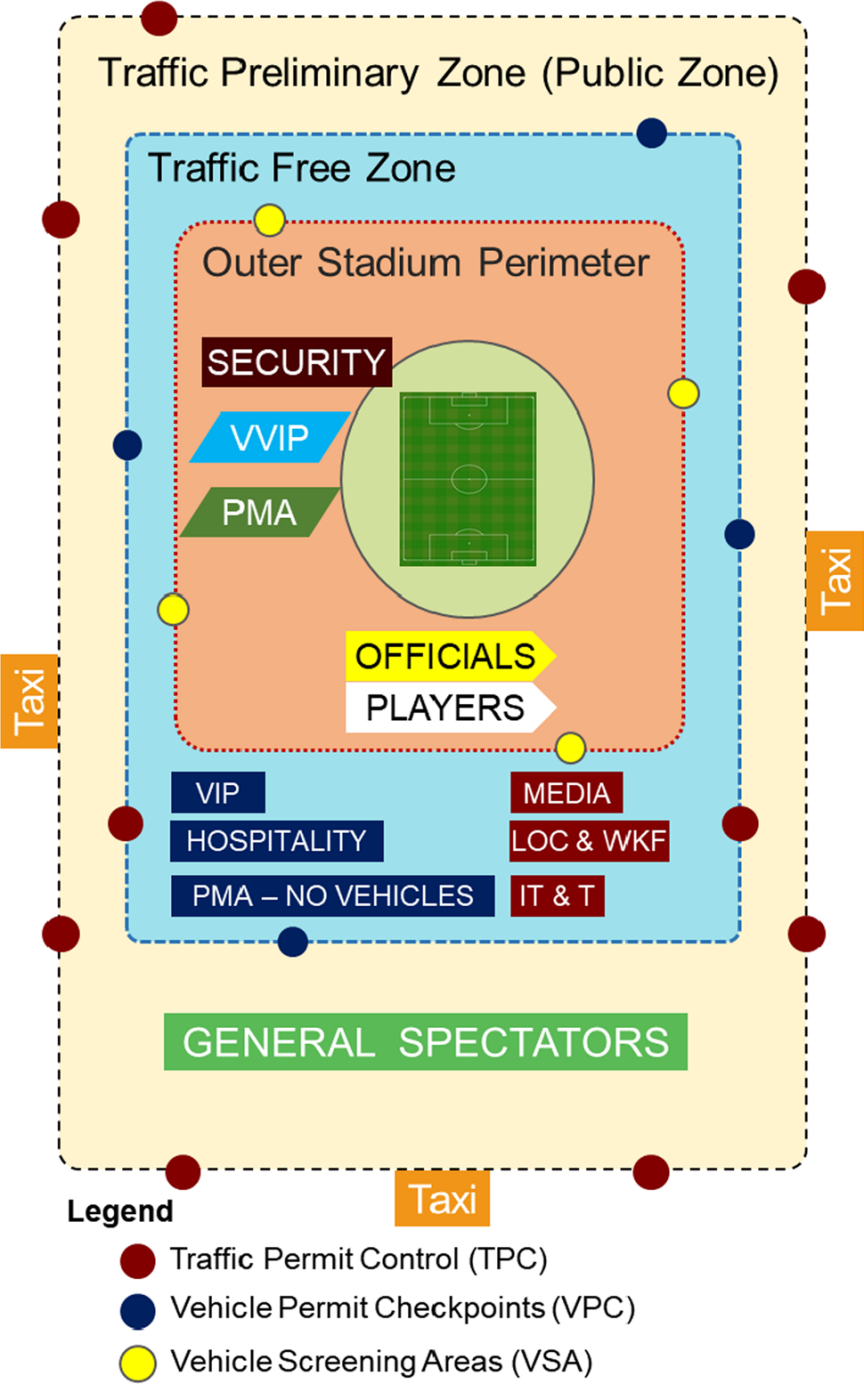
Transport general model proposed by SC for stadiums hosting FIFA World Cup 2022. SC Competition Venue Requirements: Spaces and Systems, Rev 1, May 2016

For the FIFA World Cup 2022, the accessibility strategy differs according to user groups. Figure 3 depicts a typical layout of the transport general model.

The VVIPs who are the Heads of the State and their guests will access the stadium via pre-planned routes and timings with individual security teams and coordinated with the Local Organising Committee (LOC) and the Internal Security Force (ISF) as required. The VVIPs arrivals will be staggered such that no two (2) VVIPs will arrive at the same time to avoid any security lapses.

As for the VIP spectators, they will access the stadium via private car or by VIP shuttle bus via the Traffic Free Zone (TFZ) having dedicated and secure parking within/outside but in close proximity of the secured outer stadium perimeter with easy access to the VIP reception areas. Hospitality spectators can access the stadium by private car, bus, and metro.

The players and FIFA officials arrive at the site by FIFA buses, as such, it is anticipated a shuttle bus per team plus an official bus for the referee/match officials are allocated. Expectedly this user group arrives at the ground at a specified time in accordance with LOC requirements and would need a dedicated and secured parking area within the stadium precinct.

The aforementioned user groups will be accessing the stadium via a dedicated Tournament Route Network (TRN), which is a strategic corridor providing accessibility to the stadium precinct via dedicated event lanes along this corridor. The general public will be prohibited to use these lanes and will be centrally monitored and managed by the security forces.

General Admission (GA) Spectators comprises all other spectators who do not fall into the hospitality, VIP / VVIP categories. There are four main transport modes to the stadium for General Admission Spectators, i.e.:

- Park and ride
- Metro
- Taxi
- Express coach

GA Spectators are usually not permitted to drive a private car to the stadium precinct. Instead, they will park in one of the designated park & ride facilities and access the stadium using the shuttle buses. It is anticipated that a significant proportion of the general spectators will be riding the metro by taking the Doha Metro Green Line and heading to Al Riffa Station and then walk toward the Stadium Precinct (~ 800 m). The average walking distance concerning the local weather conditions is only about 400 m as per the local guidelines. However, it is anticipated that the willingness to walk will be higher among spectators during FIFA World Cup. Given the pleasant weather condition in the months of November and December, it is expected that spectators walk on average 1 km or above to access to and egress from the precinct. In the case of Rayyan Stadium, the pedestrian bridge from the metro station is fully air-conditioned and since the match is scheduled during the winter months of Qatar, it is anticipated that spectators would be easily able to walk this distance.

In addition to the Metro, GA Spectators are also likely to arrive at the site via public transport express coaches from the Doha city center. Furthermore, it is expected that a small proportion of the spectators arrive on foot or by bike to the stadium.

The Broadcast and Information Technology and Telecommunications (IT and T) user groups may access the precinct beyond the main arrivals and departures periods and will have a minimal impact upon the spectator’s journey to the site.

The accessibility strategy needs to take a different approach for the access pattern of media. In other words, the media will require access to the Stadium while the General Admission Spectators are arriving at the site. Therefore, dedicated load zones for shuttle buses need to be located as close as possible to the hosting broadcast compound with additional on-street parking to be provided for Broadcast and Media buses once passenger drop-off has been completed.

### Public Transportation Operational Strategy

For FIFA World Cup 2022, public buses and a metro line are two major transit modes offering transportation services from and to the Al Rayyan Stadium. In this section, public transit arrangements for buses and metro during the tournament will be briefly explained.

#### Bus Operation for Al Rayyan Stadium

Generally, it is foreseen that the GA spectators will significantly utilize the public transport systems such as the metro and the buses (express coaches and shuttle buses) for the FIFA World Cup 2022. For the case of Al Rayyan Stadium, the Mode Share for the General Admission for bus and coach and metro is forecasted 67% and 40%, respectively. It should be noted that the public transit shares were provided by the Supreme Committee in this study. They were determined based on several studies to reach a sweet spot where the investment in the public transport buses, and metro capacity augmentations are justified. A further increase in public transit infrastructure may not be cost-effective.

**Fig. 4:**
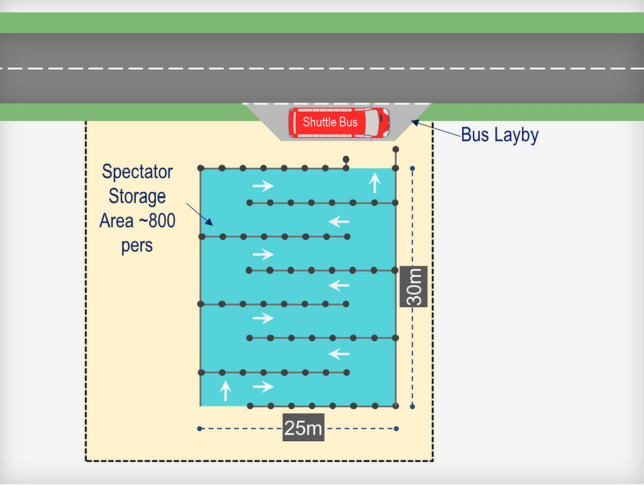
Bus stand and spectator queuing area

It is assumed that the express coaches and shuttle buses will be a 50-seater bus with an occupancy rate of 80% totaling 40 passengers. A dedicated Hospitality and GA Spectators coach pick up/drop off zones are provided within the precinct (refer to Fig. 4). The GA Spectators utilizing the Park and Ride facilities will be transported via shuttle buses to the precinct.

FIFA recommends that all the spectators shall be cleared within 90 min after the final whistle of the match. Although it is anticipated that all spectators would desire to leave the stadium immediately right after the match, in this study a uniform distribution for the spectators’ departure pattern is considered such that the infrastructure should allow for maximized peak conditions throughout the 90-min period to cater for worst traffic conditions. To achieve this, a departure headway of 3 min for shuttle buses was assumed which results in 20 coach buses per hour (60 min/3 min). The three min headway covers the thirty-second allowance for the coach bus to enter and exit the stand based on the standard operations periods, plus, the ninety-second allowance for spectators to board the coach bus with a capacity of 40 passengers per coach. An additional 1-min allowance is assumed to accommodate any further delays, which results in a four-minute headway between services.

To streamline the boarding process, each bus stand will have a Spectator Queuing Area, which is designed to hold up to 800 spectators per hour. This steady flow will be ensured by implementing crowd management measures. For a ninety-min (one hour and a half) departure clearance time, the stand would need to accommodate up to 1,200 spectators consequently. Assuming a 0.625 sq.m per passenger, each spectator queueing area is designed with an area of 800 sq.m. The below figure presents a typical layout for each bus stand along with its spectator queuing area.

#### Metro Operation for Al Rayyan Stadium

Al Rayyan Stadium has been located within walking distance of Al Riffa Metro Station—on Doha Metro’s Green Line. The metro station, Al Riffa Station, is located within a walking distance and has elevated closed walkways. This metro station will be extensively used for both Qatar and Typical Matches to transfer spectators from Doha to the Al Riffa Metro Station and vice versa. Al Riffa Station serves the Mall of Qatar as well.

The Green Line of Doha Metro at the Al Riffa station provides access to the south side of the precinct. The driverless metro cars with the interval of 2 min could offer a capacity of 8,000 passengers per hour during peak periods. It is expected that a significant amount of general admission spectators to travel by metro using the Al Riffa Metro Station which will serve Al Rayyan Stadium and Mall of Qatar. Those GA spectators will have access to the stadium on foot from the metro station to the search and screen areas of the venue.

### Travel Routes and Accessibility for Al Rayyan Stadium

Travel routes and accessibility strategies to the stadium precinct are arranged according to the user groups. The VVIPs/VIPs will use a dedicated Tournament Route Network (TRN) to access the precinct. The dedicated VVIP access route will utilize high-security measures and be protected from any public interference. To reach al Rayyan Stadium, there will be two designated routes, the primary route and the secondary route for the VVIPs. The routes will be segregated from the general public and other user groups.

The Emir, VVIPs, and VIPs are given the highest priority followed by the Match Officials and the Players, the aforementioned user groups can share the same vehicle route. However, their arrivals within the peak hours will be staggered by security operations so that no dignitaries should be waiting for the departure/arrivals of other dignitaries.

In addition to the above-mentioned user groups, Media, Local Organising Committee, Emergency Services, and Tournament Workforce will be given the priority of access and will have segregation of route from the general public. The access routes will be planned to allow accessibility of respective FIFA Constituent group vehicles through the various perimeters around the Stadium and into their relevant parking/load zone areas with minimum interactions with other user groups and pedestrians.

The spectators are placed at the base of the hierarchical chain, albeit they constitute the highest number of users. For the 2022 FIFA World cup tournament, there are two groups of spectators namely;

- Hospitality Spectators; and
- General Admission Spectators

Hospitality spectators are provided with additional complementary services included within their tickets. Hospitality spectators arriving via car to the stadium will be provided with a parking space within the precinct. Furthermore, hospitality spectators will also use the metro and hospitality tournament express coaches from the different parts of the city.

General Admission spectators (GA) will travel to the stadium precinct by a range of modes including private car, coach, Metro, taxi, and walking.

The routing options should be readily available to the public by utilizing social networks, Variable Message Signs via ITS, and other broadcast radio channels.

It is assumed the metro is utilized at its max capacity for both Qatar match and typical match scenarios, transporting a range of user groups. Spectators will walk from the metro station to the precinct, due to its proximity.

### Temporary Traffic Management (TTM) Measures, Traffic Modeling, and Mitigations for Al Rayyan Stadium

The transport strategy and the traffic inputs discussed in the previous sections are validated further by a strategic traffic model (Baseline 3 model) in PTV VISUM and at a detailed level, by a microsimulation model in PTV VISSIM. The intention is to examine the operational efficiency of the proposed road network changes and the temporary traffic management measures on mobility, accessibility, and traffic safety. The microsimulation model outputs indicate some operational deficiencies and the presence of bottlenecks, which warrant further mitigations.

Wojtowicz and Wallace (2010) emphasize the applicability of microsimulation for traffic management of special events including evacuations and incidents. They examined evacuation (egress circumstance) considering traffic management plans by incorporating improvement techniques into the current management procedures for two case studies, i.e., the evacuation of a minor-league baseball stadium, and vehicular egress plans for a large urban indoor arena in New York State’s Capital District region. Their study outcomes indicated that the real-time applicability of microsimulation in a traffic impact assessment (TIA) might bring about many new traffic management strategies.

To provide an easy and straightforward accessibility to approximately 41,000 spectators, visitors, and other user groups to the Al Rayyan Stadium and precinct during FIFA World Cup 2022 matches, implementing a set of temporary traffic management measures, restrictions and road closures will be necessary. This includes closures of main roads to the general public and implementing TPZ and VPC during the match days to cater to the spectator movement. Furthermore, imposing restrictions on left turn and right turn movements from the roads connecting to the main roads of the stadium by using temporary traffic cones is recommended.

Rerouting traffic to avoid conflicting flows for the shuttle buses and marking traffic lanes as ‘Bus Only’ lanes to provide priority to the shuttle buses are other operational tactics to improve accessibility to the stadium precinct.

For the traffic modeling assessment for the Rayyan Stadium, the Baseline 3 model provided by the SC was updated with the proposed stadium master plan along with the access arrangements. Then, the highway network amendments such as the road hierarchy, traffic analysis zones, signal controls, Park and Ride facilities road and signal capacities, temporary route arrangements, and the tournament specific restrictions, discussed earlier in this section, along with the introduction of new modes, e.g., shuttle buses and tournament express coaches, were inputted to the strategic traffic modeling software (PTV VISUM) to accurately replicate the transport strategy. The model was then run for the traffic assignments, which provides the peak hour traffic flows of the surrounding road network.

The peak hour flows were then extracted from VISUM and further operational analyses were undertaken by traffic analysis software such as Sidra (for roundabouts and right in/right outs) and SYNCHRO (for signalized intersections) to determine the Level of Service (LOS) at the surrounding junctions which range from ‘LOS A’ to ‘LOS F’. As per the applicable standards and guidelines, LOS D or above is considered acceptable for the traffic analysis.

**Fig. 5:**
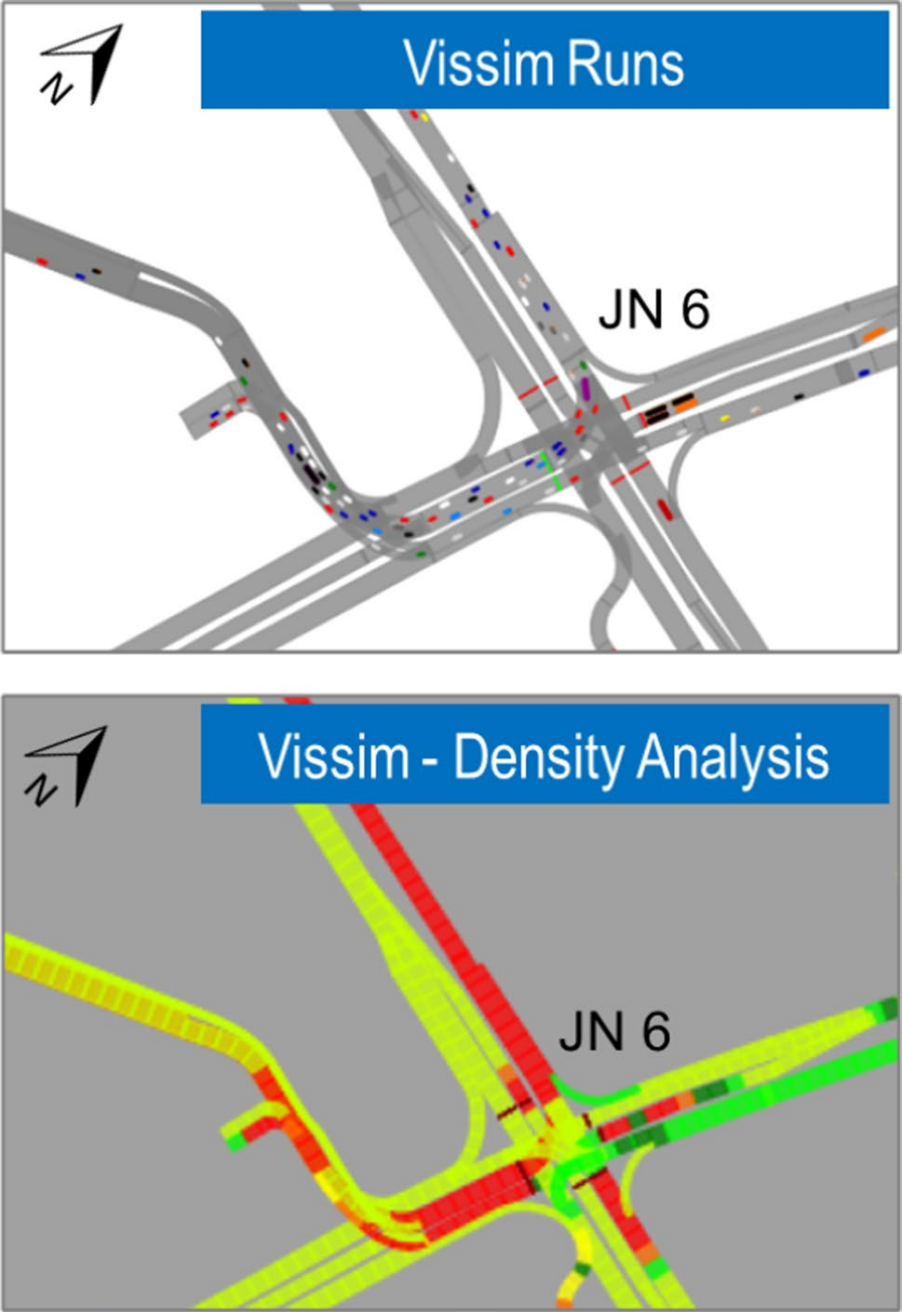
Typical intersection—VISSIM layout and LOS. Note: The link sections in red indicate traffic congestion due to high density of vehicles and the sections in the green indicate low density of vehicles and thereby less congestion

In addition to individual analysis of junctions, the entire network study area is further analyzed by VISSIM Microsimulation. To avoid any traffic-related issues during the tournament, besides the LOS criterion, visual checks in the microsimulation will be conducted to identify, if there are any localized bottlenecks, Fig. 5 shows a typical intersection modeled in VISSIM and the operational performance, which are derived visually, and by determining the LOS (based on density), respectively.

To realistically represent the traffic flow performance and to mitigate the failing junctions, a microsimulation model was built using PTV VISSIM for the Typical Match (arrival and departure scenarios) and Qatar Match (arrival and departure scenarios). These models help determine the capacity requirements of the road segments and junctions and required modifications to the transit services. The VISSIM model is primarily used as an evaluation tool to predict how well the alternative plans perform in fulfilling traffic capacity goals and thus accommodating transport demand.

The node evaluation and Level of Service (LOS) criteria for signalized intersections, links evaluation & Level of Service (LOS) criteria for links, and network density analysis are undertaken to make a better evaluation of the network performance. For the operational analysis of the intersections, Average Delay (s), LOS, Average Queue Length (m), Maximum Queue Length (m) were extracted from the microsimulation model for each match type. Based on the aforementioned analysis, it was determined and confirmed that the proposed master plan and the transport strategy will appropriately accommodate the tournament traffic for arrival and departure scenarios, with the exception of certain minor components in the modeled network, as the model demonstrated the majority of the areas within the network perform at an acceptable LOS. However, it was also observed some traffic congestions and blockages within the network, which warranted further mitigation measures such as physical and operational improvements, travel demand management strategies, or a combination of these strategies. The aforementioned measures were examined and then the best performing measures, i.e., Level of Service, operational delays, and density, were eventually recommended. The nature of the mitigations is based on their impacts on the traffic operations; for instance, only minor amendments are required to improve the traffic operations/safety at certain locations. Whereas for some other cases, a major amendment to the existing/proposed infrastructure is needed to achieve the desired traffic performance, flow rates and to ensure the safety of the road users.

Given the outcomes of the microsimulation analysis, a set of soft and hard mitigating measures are proposed based on identified traffic bottlenecks and pinch points.

The soft mitigations are temporary in nature and constitute nil to minor construction requirements. In some cases, the soft mitigations would be implemented by the use of the traffic personnel or Intelligent Transportation Systems (ITS). Some of these measures can be outlined as follows:

- Widening the turning radius,
- Implementing curbs extension,
- The provision of special road markings to provide clear information,
- Implementing partial lane closures and,
- Implementing Reversing lanes: Within the precinct parking the circulatory lanes to the parking areas will be used one directional during the pre-match (IN) and the Post-match (OUT) with an additional lane for emergency vehicle access

Besides the above-mentioned measures, some mitigations are considered as ‘Hard Mitigations’. These mitigations require medium to major amendments, which are permanent in nature. Such measures include:

- Provision of additional lanes at certain intersections and extension of storage lanes at some access points,
- The provision of median openings within the divided roads to shorten the clearance times. The turning radius is tested using the Swept Path Analysis to accommodate such maneuvers,
- Blocking conflicting movements at the surrounding junctions by using concrete barriers and thereby rerouting the traffic to take diversions; and
- In a rare case, creating an additional intersection to shorten the shuttle bus travel times to comply with the spectator’s clearance guideline of FIFA.

**Fig. 6:**
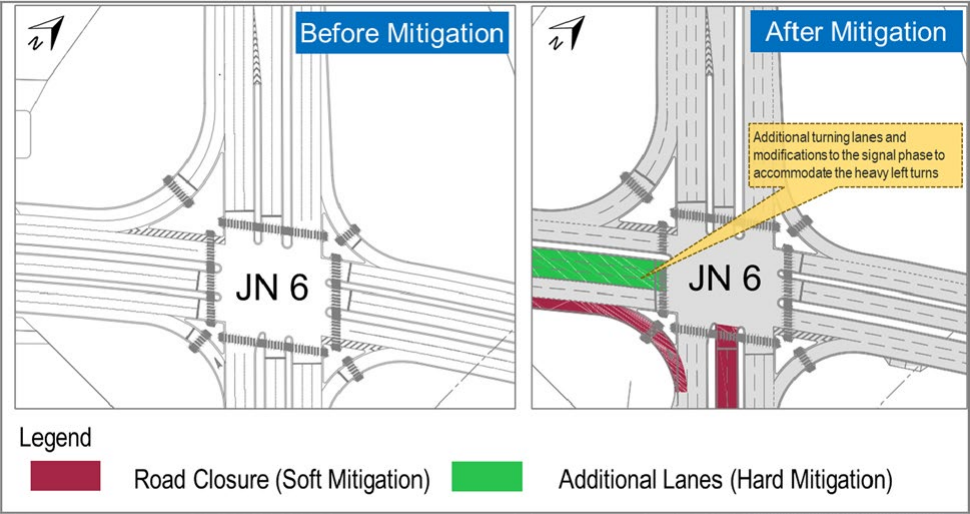
Mitigations for a typical intersection

Based on the aforementioned TTM measures and soft/hard mitigations, it was determined and confirmed by the microsimulation analysis that the proposed master plan and the transport strategy for the Rayyan Stadium will accommodate the tournament traffic demand by evaluating the operational performances against the capacity for all the scenarios. All areas within the microsimulation model were observed to be working at an acceptable LOS (LOS A to LOS D). An example of the soft mitigation and the hard mitigation applied to a typical intersection is shown in Fig. 6.

After finalizing the mitigations, a Road Safety Audit (RSA) was conducted on the overall master plan, transport strategy, and the TIA findings and recommendations. RSA is a formal examination of the operational safety of existing or future road infrastructures by an independent and qualified RSA team. It reports qualitatively on potential road safety issues and identifies opportunities to improve operational safety for all road users, with the objective of minimizing the number and severity of personal injury crashes in accordance with a safe system approach. The audit report recommendations were considered and implemented in the TIA.

**Fig. 7:**
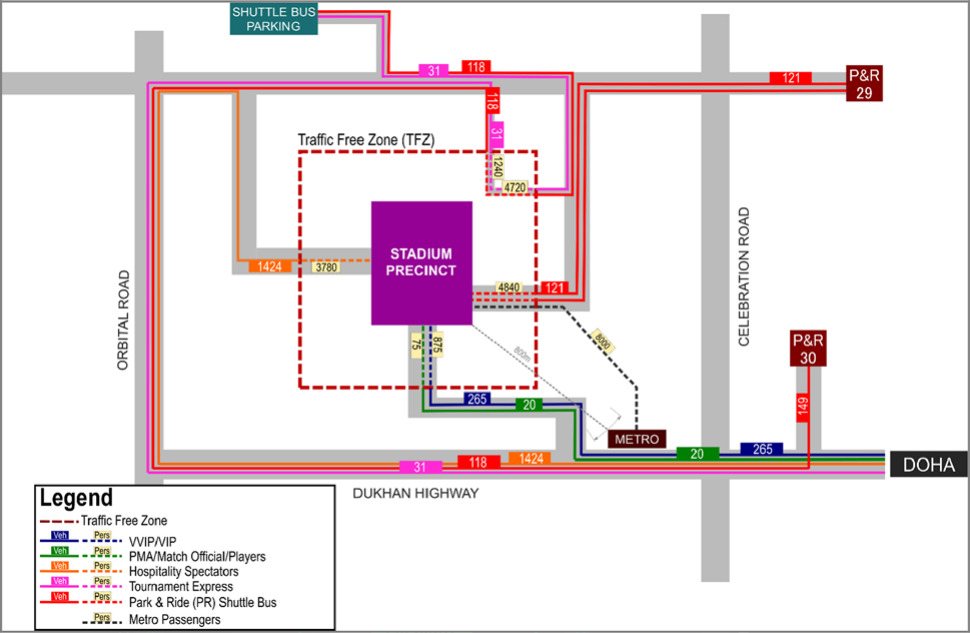
Last mile connectivity—typical schematic layout

After the completion of the RSA stage, to demonstrate the overall strategy and the flow pattern by illustrating the Last Mile Connectivity and Accessibility, a graphical map is developed. It highlights how various user groups will access the stadium precinct through their corresponding modes from different routes. The Last Mile Connectivity map also displays the information in terms of vehicles and persons for a clear understanding of the traffic flow profiles, which will be served as a crucial guide for various stakeholders such as the security specialists, police, and Internal Security Force (ISF) and is necessary for planning the tournament operations. Figure 7 depicts an example of the Last Mile Connectivity Map to demonstrate how the information is conveyed to transport, security, and enforcement authorities. As exhibited in Fig. 7, Traffic Free Zone (TFZ), Metro and Park & Ride connections to the stadium, and shuttle bus lines and routes from/toward the parking areas are specified for various types of visitors.

## Summary and Conclusions

This study centered on Traffic Impact Assessment (TIA) including the accessibility strategy and master planning for a hosting stadium for the FIFA World Cup 2022 in Al Rayyan, the State of Qatar.

The methodology applied in the TIA dealt with forecasting tournament transport demand for various user groups and workforces involved in organizing and operation the World Cup matches such as the local organizing committees, emergency services, media, and playing teams, besides general and hospitality spectators. Furthermore, requirements of the stadium precinct and the surrounding area in order to accommodate World Cup tournament demand in the context of transport master planning were discussed.

The paper also explained the accessibility strategy to the stadium precinct for each user group based on their travel mode. To do so, the access route to the stadium precinct is divided into several zones such as Traffic Preliminary Zone, Traffic Free Zone, Vehicle Checkpoints, and Vehicle Screening Area, and relevant strategies for passing through them were clarified.

A key strategy used for the planning of the FIFA tournament venues is supposed to utilize the existing infrastructure wherever possible, although this may not hold entirely true with Qatar since the majority of the infrastructure projects were built in order to cater for the upcoming tournament. Nonetheless, it is clearly understood and practiced that the provision of additional infrastructure was minimized and recommended only if absolutely necessary. This was concluded based on the findings of the traffic analysis. Rather than looking at the tournament-specific mitigations, the Traffic Impact Assessment (TIA) recommendations mainly aimed at improving the operational performance, the safety of the road users, and making efficient accessibility.

The football stadium precinct is complicated in terms of functionality, crowd management, road, and public transportation operation. The literature review presented in the article outlined some applicable arrangements particularly in the context of mobility and traffic safety for the World Cup and other major tournaments. Therefore, accessibility strategy particularly last-mile connectivity concerning safety, efficiency, and mobility concerns stemmed from huge demand, and past similar World Cup tournaments were elucidated.

To conclude, the extended traffic impact assessment discussed in this paper outlines transportation and accessibility strategy components that should be incorporated in the transportation master planning of football stadiums to comply with requirements adopted by the tournament-organizing committees such as FIFA.

The specific contributions of this paper could be outlined in the following four merits: (1) to provide a high-level overview of conducting Traffic Impact Assessment to transport & non-transport professionals, (2) to explains how to plan for the special event scenarios for traffic operations, and (3) to outline lessons learned and guidance on types of hard and soft mitigations required during large events and tournaments such as FIFA World Cup.

This study can be enriched by undertaking crowd management. During the FIFA World Cup event, which is one of the most-watched sporting events in the world, large crowds of different types can be expected not only in or around stadiums, but also at famous tourist attractions, and public transport stations such as metro, tram and bus stations. Hence, it is important to evaluate the issues, such as capacity issues, existing and potential pinch-points, and bottlenecks as for increased active modes demands. Such study should be undertaken for places where stampedes could occur when demands are suddenly increased particularly at different crowd attracting points. The intention is to make improvements to enhance safety, accessibility to and mobility within such places.

Apart from such pedestrian crowd-related issues, which are related to evacuation and capacity of the infrastructure, interactions between pedestrians and vehicles, particularly in the vicinity or outside of stadiums, park and ride spots, metro stations, and touristic attracting points will also largely influence the safety of pedestrians. Such issues should also be considered when planning public spaces for special events.

## Footnotes

1 The FIFA World Cup, is an international association football competition contested by the senior men’s national teams of the members of the Fédération Internationale de Football Association (FIFA), the sport’s global governing body.

2 FIFA, 2006, Dortmund decked out for football, retrieved from: https://www.fifa.com/worldcup/news/dortmund-decked-out-for-football-32689.

3 The Union of European Football Associations.

4 This study was concluded before the onset of Covid 19 pandemic. However, if Social distancing measures are still in place during the FIFA World Cup tournament, it is expected that the stadium capacity will reduced accordingly to comply with the social distancing measures.

## Declarations

### Data Availability

Data sharing not applicable: No new data were created or analyzed in this study. The findings in this study are based on field observation, traffic survey, and Macro and Microsimulation modeling works. Data sharing does not apply to this article.
